# Giant magnetoresistance in lateral metallic nanostructures for spintronic applications

**DOI:** 10.1038/s41598-017-09086-4

**Published:** 2017-08-25

**Authors:** G. Zahnd, L. Vila, V. T. Pham, A. Marty, C. Beigné, C. Vergnaud, J. P. Attané

**Affiliations:** SPINTEC, CEA-INAC/CNRS/Univ. Grenoble Alpes, F-38000 Grenoble, France

## Abstract

In this letter, we discuss the shift observed in spintronics from the current-perpendicular-to-plane geometry towards lateral geometries, illustrating the new opportunities offered by this configuration. Using CoFe-based all-metallic LSVs, we show that giant magnetoresistance variations of more than 10% can be obtained, competitive with the current-perpendicular-to-plane giant magnetoresistance. We then focus on the interest of being able to tailor freely the geometries. On the one hand, by tailoring the non-magnetic parts, we show that it is possible to enhance the spin signal of giant magnetoresistance structures. On the other hand, we show that tailoring the geometry of lateral structures allows creating a multilevel memory with high spin signals, by controlling the coercivity and shape anisotropy of the magnetic parts. Furthermore, we study a new device in which the magnetization direction of a nanodisk can be detected. We thus show that the ability to control the magnetic properties can be used to take advantage of all the spin degrees of freedom, which are usually occulted in current-perpendicular-to-plane devices. This flexibility of lateral structures relatively to current-perpendicular-to-plane structures is thus found to offer a new playground for the development of spintronic applications.

## Introduction

The control of multilayers growth has been a key factor for the development of spintronics. The possibility to combine ultrathin magnetic and non-magnetic layers allowed creating hetero-structures whose dimensions are smaller than the characteristic lengths of the spin-dependent transport. This has led to the discovery of the Giant Magnetoresistance^1^ (GMR), and to the development of transport experiments in the current-perpendicular to the plane (CPP) geometry.

Recent progresses in lithography techniques have enabled the creation of lateral nanodevices in which the lateral dimensions become smaller than physical lengths such as the spin diffusion length. Thus, the effects usually observed in CPP can be observed in lateral devices^2, 3^ which are nowadays widely used in spintronics. Beyond the classical magnetoresistance and spin-transfer torque effects observed in multilayers, the high flexibility of lateral geometries offers new possibilities^4^, such as separating the charge current from the spin current by using non-local measurements in lateral spin valves^2^ (LSVs).

Nevertheless, most functional spintronic applications remain based on a CPP geometry^5^, and on the use of magnetic tunnel junctions^6^. The failure to develop spintronic applications based on lateral structures is mainly due to the weakness of the spin signal amplitudes, when compared to what can be obtained in vertical devices. In the case of the giant magnetoresistance, the resistance variations in all-metallic pillars are usually of a few percent^7, 8^ (and to more than one hundred of percent using multilayers stacks^9^ or magnetic tunnel junctions^10^). As a comparison, GMR variations in LSVs are usually found to be of the order of magnitude of a percent^11, 12^, or even less, for a spin signal amplitude of a few milliohms. Nevertheless, consequent output voltages in metallic LSVs have recently been measured^3^, notably using Heuslers alloys with a high spin polarization^13–15^ or tunnel junctions^16–18^. These results are promoting the idea that the physics and technology of spin transport in lateral devices are mature enough to envision the replacement of vertical geometry by lateral architectures: for instance, the use of non-local measurement in LSVs could be applied to develop new hard disk drive read-heads^15^.

In this letter, we discuss this shift towards lateral geometries, illustrating the new opportunities offered by this configuration. Using CoFe-based all-metallic LSVs, we show that GMR variations of more than 10% can be obtained, competitive with the CPP GMR. We then demonstrate the interest of being able to tailor freely the geometries: that of the non-magnetic parts, to enhance the signal, and that of the magnetic parts, to control the magnetic properties, to create memory functionalities, or to take further advantage of the spin degrees of freedom.

**Figure 1:**
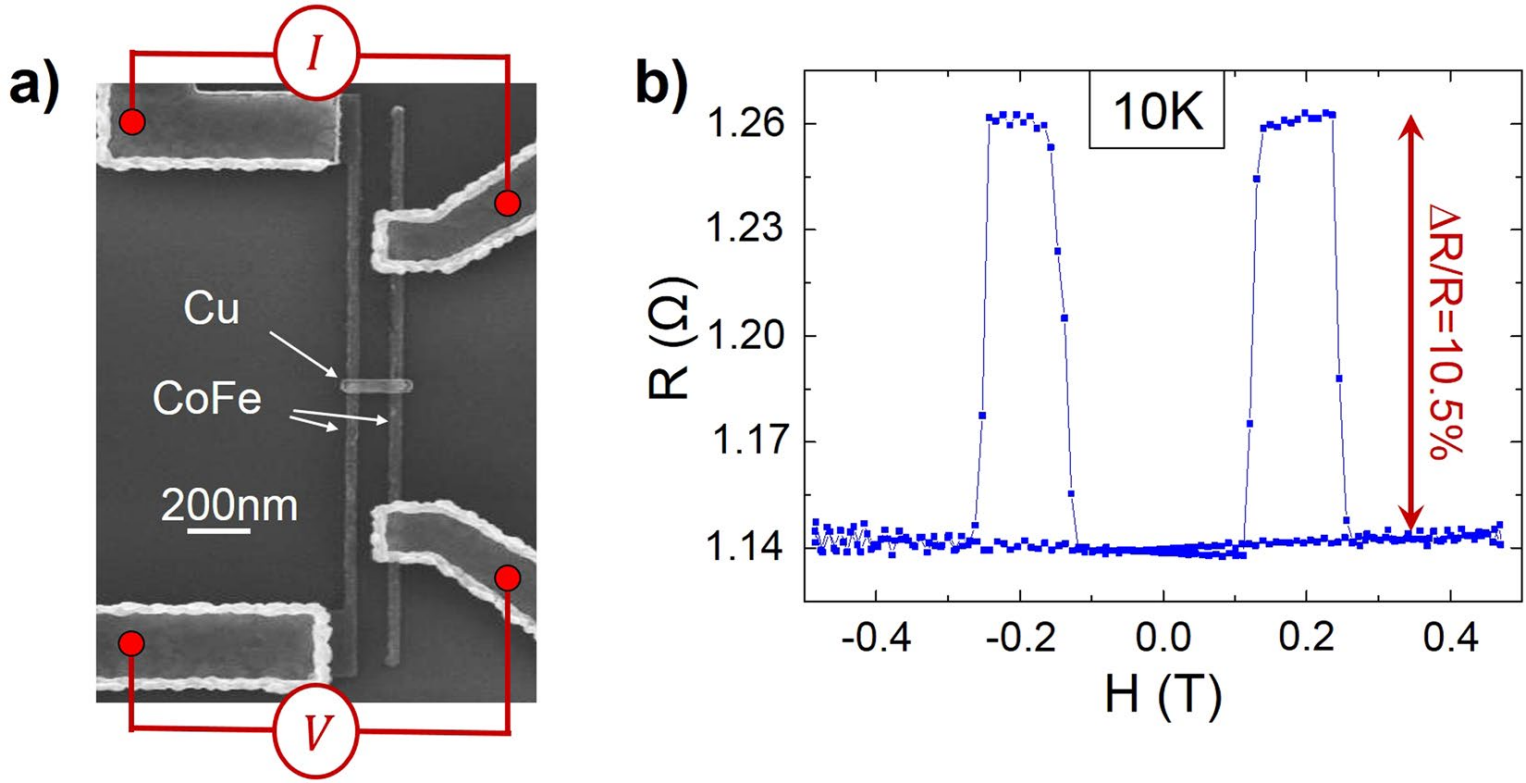
(**a**) SEM image of a CoFe-based lateral spin valve, with the electrical setup for GMR measurements. The inter-electrode gap is 150 nm wide from centre to centre (*i.e*. 100 wide from edge to edge). The non-magnetic channel is made of Cu. All nanowires are 50 nm wide. CoFe wires are 15 nm thick, while Cu wires are 80 nm thick. (**b**) Corresponding GMR measurement at 10 K, obtained with a magnetic field parallel to the ferromagnetic electrodes. The relative resistance variation is ΔR/R = 10.5%.

Let us firstly focus on how the spin signal amplitudes of all-metallic lateral devices, once optimized, can compete with multilayer GMRs. CoFe/Cu LSVs have been nanofabricated, with a very short spin channel to avoid spin relaxation in the Cu. Note that this non-magnetic channel has been restricted to the central part of the LSV only^19^ to avoid additional relaxation on the side (see Fig. 1a or the supplementary materials). Among several other ways to increase spin signal amplitudes^13–17^, it has indeed been proposed to use CoFe electrodes in LSVs, in replacement of the widely used NiFe electrodes^20^. GMR measurements can be performed in LSVs, using the electrical probing setup of Fig. 1a. GMR variations of 5.8% are obtained at room temperature, and more than 10% at 10 K, as seen in Fig. 1b. These values have to be compared to GMR variations obtained using CPP measurements in equivalent nanopillars, e.g., in CoFe/Cu/CoFe^7^ or Co/Ni_4_/Cu/Co/Ni_4_^8^, which are found to be smaller (2.5% and 3.5%, respectively). This competitiveness of lateral devices pleads for their use in spintronics applications, knowing that further amplification means can be used, such as the use of magnetic tunnel juctions^16^ or Heuslers alloys^15^. Also, according to the 1D analytical expression based on the Valet Fert model^2^, reducing the dimensions of the wire widths leads to the signal enhancement. Indeed the spin signal in the GMR configuration can be written^19^:

1 $${{\rm{\Delta }}{\rm{R}}}_{{\rm{GMR}}}=\frac{8{{{\rm{P}}}_{{\rm{F}}}}^{2}{{\rm{R}}}_{{\rm{F}}}^{\ast 2}\,{{\rm{R}}}_{{\rm{N}}}}{{({{\rm{R}}}_{{\rm{N}}}+2{{\rm{R}}}_{{\rm{F}}}^{\ast })}^{2}{{\rm{e}}}^{\frac{{\rm{L}}}{{{\rm{\lambda }}}_{{\rm{N}}}}}-{{\rm{R}}}_{{\rm{N}}}^{2}{{\rm{e}}}^{-\frac{{\rm{L}}}{{{\rm{\lambda }}}_{{\rm{N}}}}}}$$

where ${{\rm{R}}}_{{\rm{N}}}=\frac{{{\rm{\rho }}}_{{\rm{N}}}{{\rm{\lambda }}}_{{\rm{N}}}}{{{\rm{A}}}_{{\rm{N}}}}$ and ${{\rm{R}}}_{{\rm{F}}}^{\ast }=\frac{{{\rm{\rho }}}_{{\rm{F}}}{{\rm{\lambda }}}_{{\rm{F}}}}{(1-{{\rm{P}}}_{{\rm{F}}}^{\,\,\,2}){{\rm{A}}}_{{\rm{F}}}}$ are the spin resistances of the materials, and where ρ_i_, λ_i_, P_i_, A_i_, w_i_ and t_i_ are the resistivity of the i^th^ material, its spin diffusion length, its polarization (for a ferromagnetic material), the effective section, the width and thickness of the wires, respectively. L is the inter-electrode distance. Since ${{\rm{A}}}_{{\rm{N}}}={{\rm{w}}}_{{\rm{N}}}{{\rm{t}}}_{{\rm{N}}}$ and ${{\rm{A}}}_{{\rm{F}}}={{\rm{w}}}_{{\rm{N}}}{{\rm{w}}}_{{\rm{F}}}$, and by considering ${{\rm{R}}}_{{\rm{F}}}^{\ast }\ll {{\rm{R}}}_{{\rm{N}}}$, it can be pointed out that^21^:

2 $${{\rm{\Delta }}{\rm{R}}}_{{\rm{GMR}}}\, \sim \frac{4{{{\rm{P}}}_{{\rm{F}}}}^{2}\,{{\rm{R}}}_{{\rm{F}}}^{\ast 2}}{{{\rm{R}}}_{{\rm{N}}}\,\sinh (\frac{{\rm{L}}}{{{\rm{\lambda }}}_{{\rm{N}}}})}\propto \,\frac{{{\rm{t}}}_{{\rm{N}}}}{{{\rm{w}}}_{{\rm{N}}}{{\rm{w}}}_{{\rm{F}}}^{2}}$$

This implies that a decrease by a factor two of the wire width should lead to an eight times increase of the spin signal, and a four times increase of the GMR ratio. Also, reducing the inter-electrode gap should linearly decrease the resistive offset, while increasing the spin signal.

Let us now focus on the geometry of the non-magnetic spacer. In a classical GMR multilayer, the only geometrical parameter of the non-magnetic layer is its thickness, which has to be as small as possible to avoid spin relaxation. In a CPP configuration, increasing the spacer material volume, thus its thickness, will immediately induce a decrease of the spin signal.

**Figure 2:**
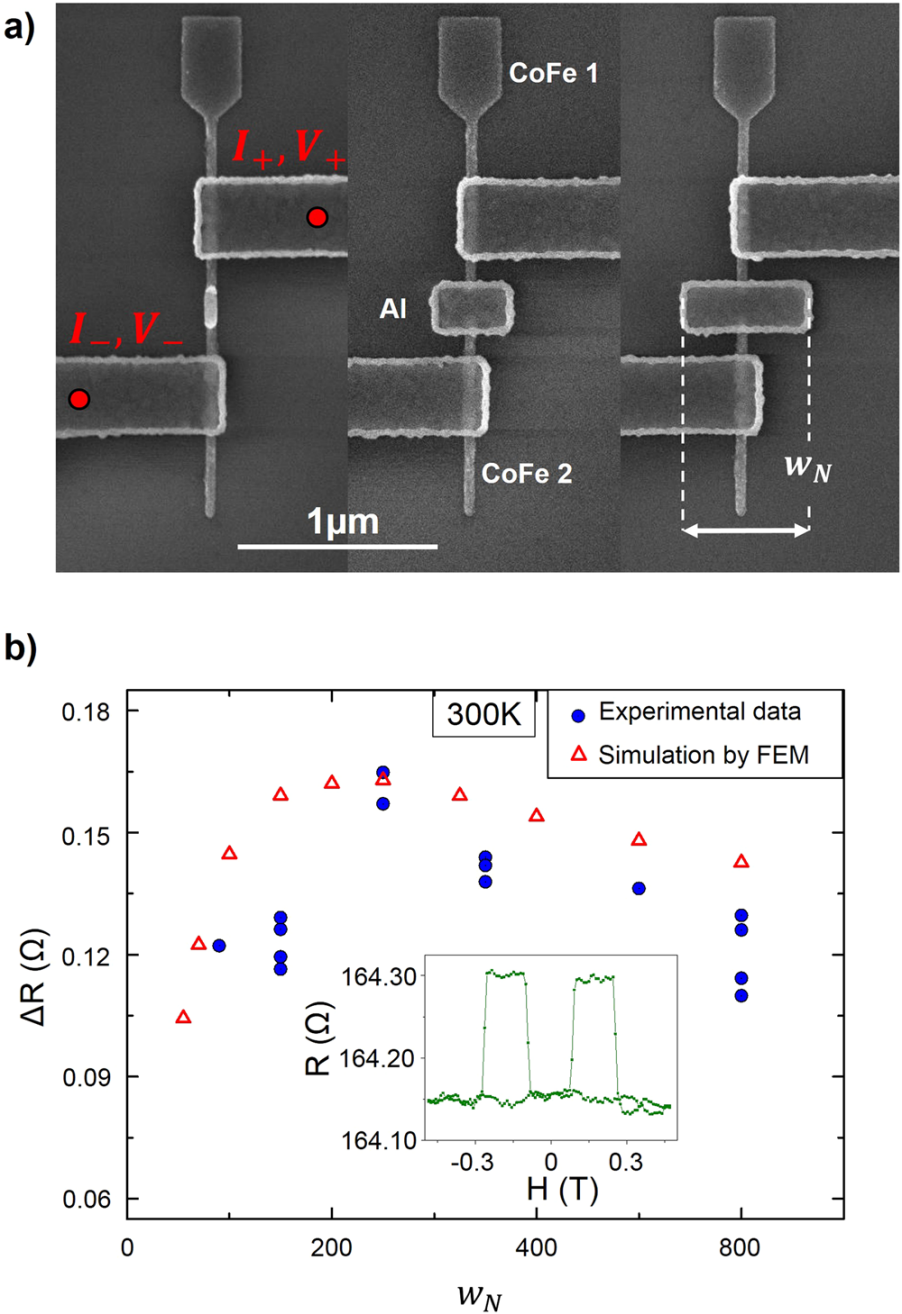
(**a**) SEM images of several CoFe/Al/CoFe GMR nanostructures, on which the resistance is measured using a two probes measurement configuration. The non-ferromagnetic part in-between the ferromagnetic electrodes 1 and 2 are rectangles of various widths, acting similarly to a spacer in a classical CPP GMR stack. For all devices, the thicknesses of the layers, the areas of contacts and the distances between electrodes CoFe 1 and 2 are kept identical. (**b**) Evolution of the spin signal amplitude as a function of the width of the rectangular spacer. Each blue dot corresponds to a single device measured. The red triangles corresponds to the results of 3D simulations done using a finite element method. Inset: example of spin signal measured in the two probes configuration, for a device with a 350 nm wide spacer.

In lateral devices, a new degree of freedom is opened, as the non-magnetic part can be tailored at will. GMR devices have been created with a geometry equivalent to that of a CPP GMR nanopillar (cf. Fig. 2a and in the supplementary materials for more details). Cu is a choice material to maximize the GMR ratio, since its resistivity is very low. Nevertheless, Al has been used in the following, as CoFe/Al-based nanostructures provide higher spin signals than Cu/CoFe-based ones^20^.

The distance between the CoFe electrodes (1 and 2 in Fig. 2(a)), which would correspond to the thickness of the spacer in a CPP experiment, is kept constant (100 nm), as well as the contact areas between the electrodes and the spacer.

In these lateral devices, it is possible to vary the width and thickness of the rectangular spacer. Note that the design corresponds to a simple two probe geometry, equivalent to that of a CPP GMR nanopillar. Although the GMR ratio is not optimized (the long and resistive CoFe ferromagnetic nanowires lead to large resistive offsets), the spin signal is very large for a GMR structure (up to 0.163 Ω at 300 K), allowing the study of these new degree of freedom.

By analogy with CPP GMR, and because of the relaxation occurring in the non-ferromagnetic material, one could suppose that an increase of the spin signal amplitude could be obtained simply by decreasing the spacer dimensions. To investigate that point, we tailored several devices with varying widths of the aluminium spacers (see Fig. 2a). The evolution of the spin signal as a function of the spacer width is displayed in Fig. 2b. The maximum is not obtained for the smallest spacer, but rather surprisingly appears at a large width of 250 nm (for a signal of 0.163 Ω), which corresponds to a width-over-length-ratio exceeding one.

This behaviour can be reproduced by simulations (cf. Fig. 2b) performed using finite element method, and based on a two spin-current drift-diffusion model^22^. These simulations have been performed using GMSH^23^ for the geometrical construction, the meshing, and the post-processing part, and GETDP^24^ as its associated solver (See ref. 25 or in the supplementary materials for details on the simulation method). The resistivities and spin diffusion lengths of CoFe and Aluminium are taken as 28 μΩ.cm, 6 μΩ.cm, 3.5 nm and 550 nm, respectively. The polarization of CoFe is set at 0.58. These material parameters are the same as those used ref. 20, where they had been extracted by studying the gap dependence of the spin signal.

The existence of this optimum can be understood by considering the competition of two antagonist mechanisms. At low widths, by the mean of a 1D analytical model^26^, one can obtain the following expression for the spin signal:

3 $${{\rm{\Delta }}{\rm{R}}}_{{\rm{GMR}}}=\frac{4{{{\rm{P}}}_{{\rm{F}}}}^{2}{{\rm{R}}}_{{\rm{F}}}^{\ast {\rm{2}}}}{2{{\rm{R}}}_{{\rm{F}}}^{\ast }\cosh (\frac{{\rm{L}}}{{{\rm{\lambda }}}_{{\rm{N}}}})+\frac{({{\rm{R}}}_{{\rm{F}}}^{\ast 2}+{{\rm{R}}}_{{\rm{N}}}^{2})}{{{\rm{R}}}_{{\rm{N}}}\,}\,\sinh (\frac{{\rm{L}}}{{{\rm{\lambda }}}_{{\rm{N}}}})} \sim \frac{4{{{\rm{P}}}_{{\rm{F}}}}^{{\rm{2}}}{{\rm{R}}}_{{\rm{F}}}^{\ast 2}}{2{{\rm{R}}}_{{\rm{F}}}^{\ast }\cosh (\frac{{\rm{L}}}{{{\rm{\lambda }}}_{{\rm{N}}}})+{{\rm{R}}}_{{\rm{N}}}\,\sinh (\frac{{\rm{L}}}{{{\rm{\lambda }}}_{{\rm{N}}}})}$$

Using the notation of equation (1). Since ${{\rm{A}}}_{{\rm{N}}}={{\rm{w}}}_{{\rm{N}}}{{\rm{t}}}_{{\rm{N}}}$, an increase of the width will induce a decrease of the spin resistance of the spacer. As can be seen in equation (3), and conformingly to several studies of the spin transport properties in lateral structures^19, 27^, optimizing the spin signal amplitude requires increasing the ratio $\frac{{{\rm{R}}}_{{\rm{F}}}^{\ast }}{{{\rm{R}}}_{{\rm{N}}}}$ to limit the spin resistance mismatch. In our case, increasing the width of the spacer lowers R_N_, diminishes the backflow, and thus leads to an increase of the signal. It is interesting to note that the 1D model also predicts the existence of a maximum spin signal, obtained for $\frac{{{\rm{R}}}_{{\rm{F}}}^{\ast }}{{{\rm{R}}}_{{\rm{N}}}} \sim 1$. This corresponds to ${{\rm{w}}}_{{\rm{N}}} \sim 700\,{\rm{nm}}$ (more details in the supplementary materials), which is much larger than the obtained optimum (by simulation and experiment) found to be W_N_ = 250 nm. Indeed for large spacers the 1D approximation becomes invalid, as the spin diffusion in the lateral directions becomes important. Widening the spacer will increase the volume of the non-ferromagnetic material in which the spin accumulation will relax, hence leading to a decrease of the spin signal.

The simplest geometry, corresponding roughly to a CPP GMR Pillar, would be that of a device with wires of CoFe and Al of equal widths. This geometry, which one could naïvely supposed to be the one giving the best spin signal, is actually quite different from the non-intuitive, optimized geometry, with a spacer width of 250 nm instead of 50 nm. Note that the geometry optimization requires 3D simulations, and cannot be done simply using 1D models. Also, the effect of the optimization is quite large, as the optimized geometry leads to an increase of more than 30% of the spin signal. Note also that in order to maximize the spin signal, the length of the spacer would have to be as small as possible, similarly to what can be observed in lateral spin-valves^2^. In that sense, the ability to tailor freely the geometry in lateral structures offers a new playground, richer than what could be done with classical multilayers.

**Figure 3:**
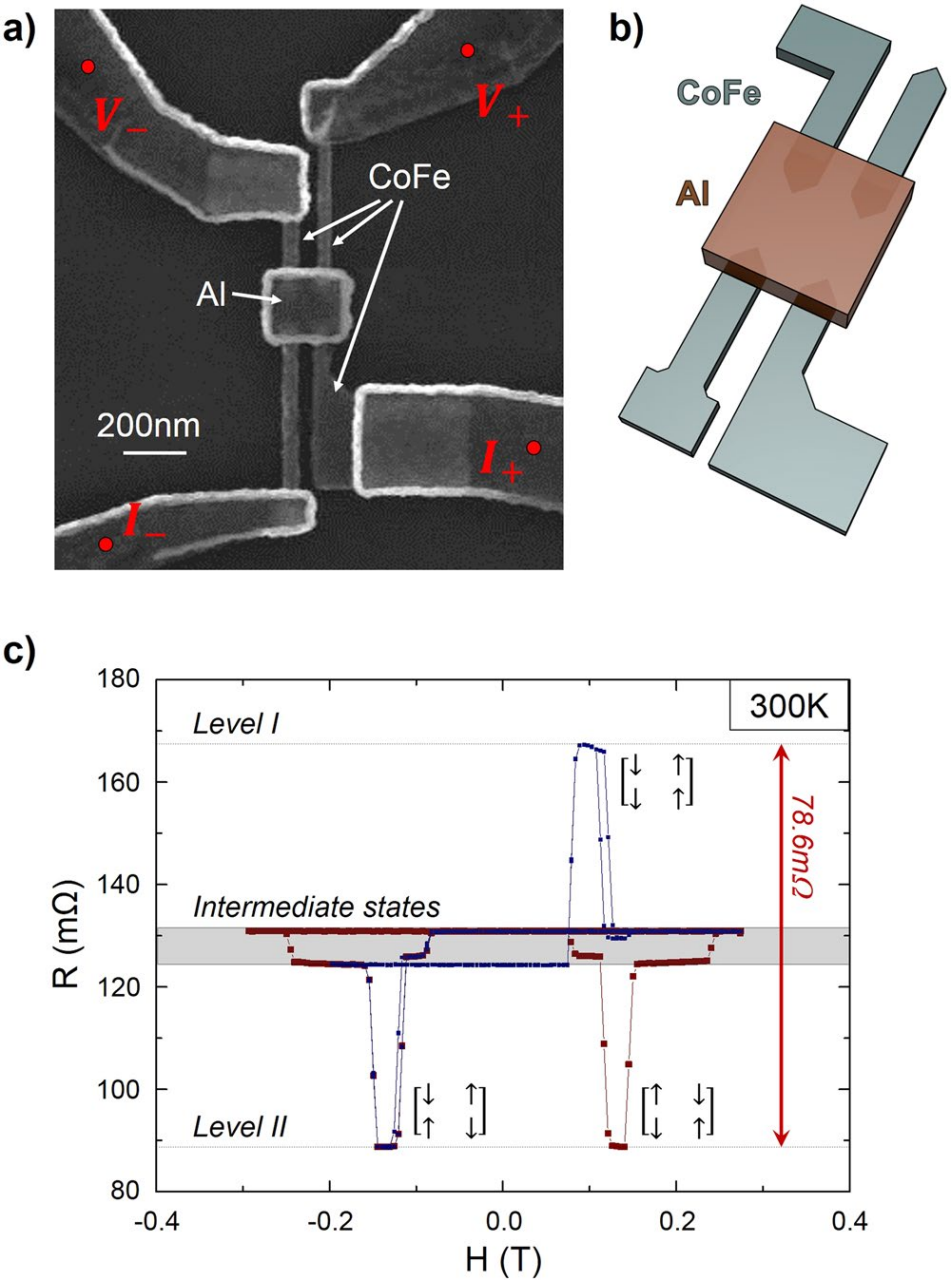
(**a**) SEM image of a multi-terminal device. The four CoFe magnetic electrodes are connected to an Al spacer. Each electrode has a distinct switching field. Different electrical setups for GMR measurements are possible, only one of them is presented here. The electrodes through which the current goes are used to create a spin accumulation. The two other electrodes are voltage probes, the voltage differences being due to both the Ohmic resistance and the spin accumulation. (**b**) Schematic illustration of the sample, enabling to distinguish more clearly the geometry of the ferromagnetic elements located beneath different non-magnetic parts (**c**) Two GMR measurements obtained in the measurement configuration of fig. (**a**) at 300 K. The red curve correspond to a field loop reaching saturation, while the blue one is a minor loop. Due to symmetries of the geometry (the contact areas and inter-electrodes distances are identical), the spin signal level are degenerated with respect to the magnetic states. As an example, the spin signal of level I corresponds actually to two magnetic configurations, as does the spin signal of level II. The four intermediate levels of signal (in the grey area) gather 12 magnetic states.

By reducing the constraints on the circuit design, the use of lateral geometries allows tailoring the non-ferromagnetic parts. It also allows tailoring the geometry of the ferromagnetic parts, which can be used in order to control their magnetic properties in a way that is impossible in multilayer structures. The geometry of the ferromagnetic elements can be used to control their coercivity^28, 29^, their shape anisotropy^30^, or the behaviour of domain walls^31, 32^. As an example, let us study a simple multi-level GMR device. This nanostructure, presented in Fig. 3a, then consists in four ferromagnetic wires connecting a central Aluminium square. The ferromagnetic electrodes possess different nucleation pads, so that they possess distinct magnetization switching fields. By doing minor loops, it is possible to set the device in any of the 16 magnetic configurations.

The electrical setup used to measure the GMR of the device is presented in Fig. 3a and b. The current flowing through the CoFe/Al/CoFe circuit generates a spin accumulation, which is probed by the two other electrodes. Figure 3c display magnetoresistance curves measured in this electrical setup at 300 K. The total spin signal amplitude (between the levels I and II on Fig. 3c) is 78.6 mΩ. A first interesting point is that, contrarily to the two probe geometry presented in Fig. 2, the design allows avoiding part of the Ohmic drop, similarly to non-local measurements in lateral spin-valves^33, 34^. The background resistance is thus reduced from a few Ohm to less than one hundred milliohms, and the observed resistance variation is very high, exceeding 88%. This geometry could be of interest for noise reduction, and depending on the required output level tunnel junctions could be inserted to enhance the signal^16^.

A second point of interest appears when performing different minor loops, in order to reach different magnetic configurations. Each configuration corresponds to a particular spin signal level. These levels are two fold degenerated, as by symmetry the reversal of all magnetizations leads to an identical spin signal. For instance, if one represents the magnetization direction of the four electrodes using arrows, the magnetic configurations $$[\begin{array}{cc}\uparrow  & \uparrow \\ \uparrow  & \downarrow \end{array}]$$ and $$[\begin{array}{cc}\downarrow  & \downarrow \\ \downarrow  & \uparrow \end{array}]$$ give identical spin signals. When the magnetizations of the injecting electrodes are parallel ($$[\begin{array}{cc}\uparrow  & \downarrow \\ \downarrow  & \downarrow \end{array}]$$, $$[\begin{array}{cc}\uparrow  & \uparrow \\ \uparrow  & \uparrow \end{array}]$$, $$[\begin{array}{cc}\uparrow  & \uparrow \\ \downarrow  & \downarrow \end{array}]$$, etc.) the spin accumulation is small, and the spin signal is nearly zero. When the detecting electrodes are parallel ($$[\begin{array}{cc}\uparrow  & \uparrow \\ \downarrow  & \uparrow \end{array}]$$, $$[\begin{array}{cc}\downarrow  & \downarrow \\ \uparrow  & \uparrow \end{array}]$$, $$[\begin{array}{cc}\downarrow  & \downarrow \\ \uparrow  & \downarrow \end{array}]$$, etc.), they roughly probe the same electrochemical potential, and the spin signal is here again close to zero.

There remain four states, corresponding to only two high values of spin signals: one of positive value ($$[\begin{array}{cc}\uparrow  & \downarrow \\ \uparrow  & \downarrow \end{array}]$$ or $$[\begin{array}{cc}\downarrow  & \uparrow \\ \downarrow  & \uparrow \end{array}]$$, level I on Fig. 3c), one negative ($$[\begin{array}{cc}\uparrow  & \downarrow \\ \downarrow  & \uparrow \end{array}]$$ or $$[\begin{array}{cc}\downarrow  & \uparrow \\ \uparrow  & \downarrow \end{array}]$$, level II on Fig. 3c). This nanostructure can thus be considered as a three level memory device. Note that additional levels could be easily obtained by lifting the degeneracy, for instance by patterning different contact areas for the different electrodes.

In this device, it can be seen that the geometrical flexibility associated to lateral structures allows controlling the properties of the ferromagnetic elements, and getting rid of the background resistance of CPP nanostructures. Let us show in the following that it also becomes possible to take advantage of the several degrees of freedom of the spin.

**Figure 4:**
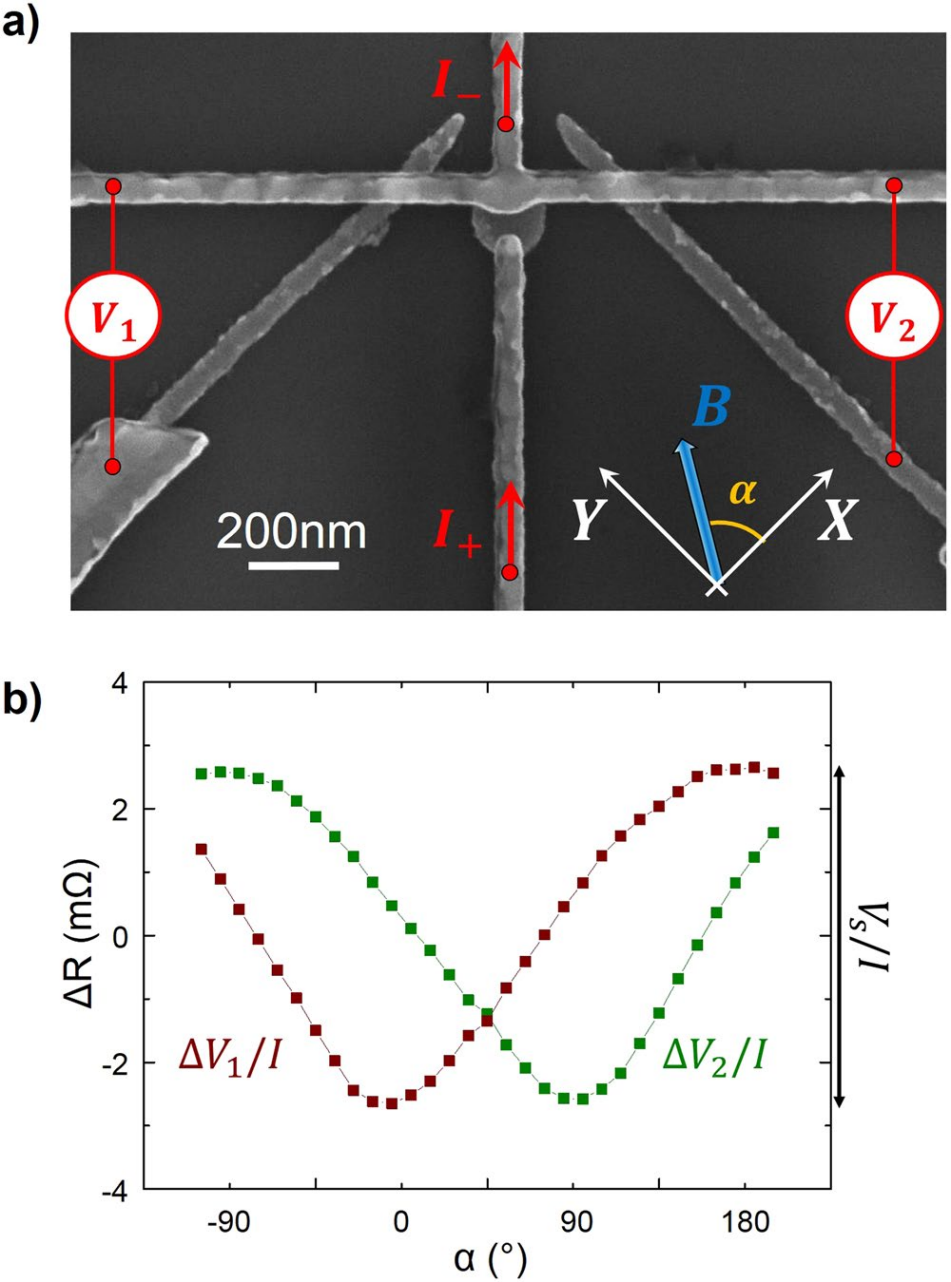
(**a**) SEM image of a setup allowing to detect the orientation of the magnetization direction of a CoFe nanodisk by two orthogonal CoFe wires. Al is used for the nonmagnetic parts. The current is injected through the nanodisk, while two non-local detectors are measuring the non-local spin signal *V*_1_ and *V*_2_. The in-plane orientation of the field is parametrized by the angle α. (**b**) Spin signals vs. field orientations α at 300 K. The applied field is kept constant in amplitude (B = 180 mT). As the magnetization of the nanodisk follows the field, the spin accumulation orientation varies. The voltage measurements using the two perpendicular CoFe wires allows measuring the projection of this spin accumulation on the X, Y basis.

Using the GMR or TMR in a CPP geometry, the magnetization direction cannot be known unambiguously. The nanodevice shown in Fig. 4a enables detecting electrically the magnetization direction of a nanodisk: each component of the spin accumulation can be probed independently.

The central ferromagnetic element is a CoFe nanodisk, 160 nm in diameter, and 15 nm thick. Two CoFe electrodes, 50 nm wide and 15 nm thick, are oriented along the X and Y directions, in a configuration similar to what was used by Kimura *et al*. to control the orientation of injected spins^35^. Al is used to connect the nanodisk and the ferromagnetic electrodes.

An applied field is used to transform the ground magnetization state of the nanodisk, a vortex, into a saturated state (obtained for B > 110 mT according to OOMMF^36^ simulations). When injecting a current through the magnetically saturated nanodisk, a spin accumulation is produced, which diffuses in the horizontal non-magnetic channel towards both the lateral electrodes.

Measurements have been performed by applying a magnetic field of 0.18T with a varying orientation *α*. The nanodisk being saturated, the direction of the majority spins within the spin accumulation is parallel to the magnetic field. Each electrode detects the component of the spin accumulation along its magnetization direction, *i.e*., X or Y. One thus measures the components *M*_*X*_ and *M*_*Y*_ of the nanodisk magnetization along the X and Y directions:

4 $$\frac{{V}_{1}}{{V}_{s}}=-cos(\alpha )=\frac{{M}_{X}}{{M}_{s}}$$

4’ $$\frac{{V}_{2}}{{V}_{s}}=-sin(\alpha )=\frac{{M}_{Y}}{{M}_{s}}$$

where *V*_1_ and *V*_2_ are the measured voltages, *V*_*S*_ is the amplitude of the voltage variations, and ${M}_{s}$ is the saturation magnetization of the nanodisk.

The spin signals are recorded as a function of the orientation $\alpha$ of the magnetic field, so that according to eq. 4 sinusoidal variations are expected. Figure 4b exhibits the obtained spin signal, which corresponds well to the expected behaviour. Note that the signals are slightly distorted sinusoidal curves: because of the field applied during the measurement, the magnetizations of the probing electrodes do not remain exactly parallel to the easy axis orientations X and Y.

Note also that one could imagine using such kind of device to perform spin transfer torques experiments: the external electrodes could serve both to create the spin transfer torque and to read the magnetization direction of the nanodisk. It would then offer the possibility to inject spins with the desired orientation, and to maximize the torque.

To sum up, we studied in this letter the shift from multilayers to lateral structures in spintronics. Beyond the fact that spin signals obtained in purely metallic nanostructures can compete with CPP GMR, we illustrated this shift by evidencing the new opportunities offered by lateral geometries. In particular we demonstrated the interest of being able to tailor freely the geometries. On one hand, the tailoring of the non-magnetic parts allows enhancing the spin signal of GMR structures. On the other hand, the tailoring of the magnetic parts allows controlling the coercivity and the shape anisotropy of the magnetic parts, creating multilevel memories, or taking advantage of the spin degrees of freedom to detect the components of the magnetization along several directions.

The lateral geometry has an associated drawback, the device size, which might restrain lateral devices to applications such as read-heads, or in which the main issue is the non-volatility or the reliability rather than the density. However, the use of vertical interconnect accesses to contact the active part, or the development of 3D stacking^37, 38^ should help solving that issue.

In any case, the flexibility of lateral structures can be combined to the use of spin-orbit effects to create and detect spin accumulations, and to the use of spin-transfer torques to switch magnetizations, thus offering a new playground for the development of spintronic applications.

## Method

All the devices have been patterned by e-beam lithography on a SiO_2_ substrate. Ferromagnetic nanowires have been fabricated by evaporation of pellets through a patterned PMMA resist mask and subsequent lift-off. In a second lithography step, the non-magnetic channels have been realized using the same lithography, evaporation and lift-off process. An Argon ion beam milling has been used *in-situ*, before the evaporation step, in order to obtain clean transparent interfaces between the ferromagnetic and non-magnetic wires.

Measurements have been performed using lock-in techniques, with an applied current of 100 µA at 488 Hz.

The datasets generated during and/or analysed during the current study are available from the corresponding author on reasonable request.

## Electronic supplementary material

Supplementary materials
