# Supplementary material for: Giant magnetoresistance in lateral metallic nanostructures for spintronic applications
Source: Sci Rep. 2017 Aug 25;7:9553. doi: 10.1038/s41598-017-09086-4 (PMC5573406; doi:10.1038/s41598-017-09086-4)
Supplement: Supplementary file 1 — Supplementary materials [file 41598_2017_9086_MOESM1_ESM.pdf]

Giant magnetoresistance in lateral metallic nanostructures for spintronic applications

SUPPLEMENTARY MATERIALS

G. Zahnd[1], L. Vila[1,] , V.T. Pham[1], A. Marty[1], C. Beigné[1], C. Vergnaud[1] and J. P. Attané[1,†]*

[1] SPINTEC, CEA-INAC/CNRS/Univ. Grenoble Alpes, F-38000 Grenoble, France

LATERAL SPIN VALVE GEOMETRY

Figure S1 describes further the nanodevice presented in the figure 1 of the manuscript. The ferromagnetic nanowires are 50n wide (w_F) and 15nm thick (t_F). The non-ferromagnetic nanowire is 50nm wide (w_F) and 80nm thick (t_F). The distance inter-electrodes (L) is 100nm edge-to-edge.

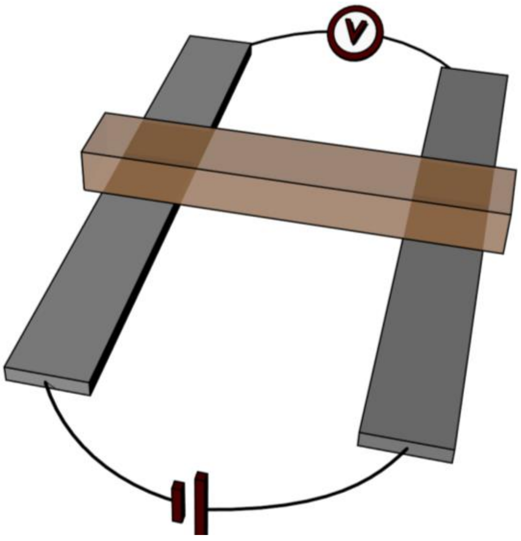

Figure S1: Schematic the CoFe and Cu parts of the device. The electrical setup for GMR measurements is also represented.

* Laurent.vila@cea.fr
† Jean-philippe.attane@cea.fr

WIDTH DEPENDANCE STUDY

Figure S2 describes further the set of nanodevices presented in the figure 2 of the manuscript. The ferromagnetic nanowires are 50nm wide (w_F) and 20nm thick (t_F). The overlap with the non-magnetic spacer is 50nm long (d_F). Note that the last 25nm of the wires are of triangular shape, in order to limit domain wall nucleation and to avoid inhomogeneous magnetization at the edges of the wires. The contact areas are thus $A_F = \frac{3}{4} w_F d_F$. The distance inter-electrodes (L) is 100nm, while the length of the spacer is 200nm. Its thickness (t_N) is 60nm, while the width (w_N) is the varying parameter.

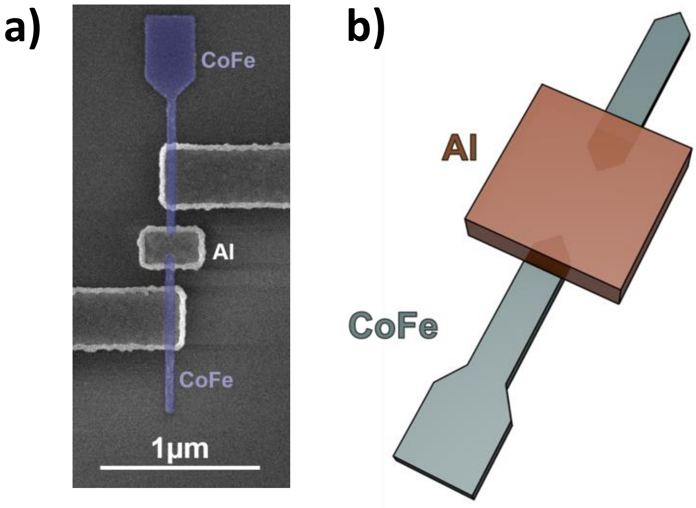

Figure S2: a) SEM image of a nanodevice with a 350nm wide spacer. The ferromagnetic elements are colored in blue. b) Schematic the CoFe and Al parts of the device.

As described in the manuscript, the optimum GMR spin signal is expected for $\frac{R_F^*}{R_N} \sim 1$ according to the 1D model.

Since $R_N = \frac{\rho_N \lambda_N}{w_N t_N}$ and $R_F^* = \frac{1}{1-P_F{}^2} \frac{\rho_F \lambda_F}{\frac{3}{4} w_F d_F}$ this condition is fulfilled for:

$$w_N = \frac{\rho_N \, \lambda_N \, (1 - P_F{}^2)}{\rho_F \, \lambda_F} \frac{3 \, d_F \, w_F}{4 \, t_N} \sim 700nm$$

This value is much larger than the obtained optimum (by simulation and experiment) found to be $w_N = 250$nm. Indeed for large spacers the 1D approximation becomes invalid, as the spin diffusion in the lateral directions becomes important.

MULTILEVEL SYSTEM GEOMETRY

The nanodevice presented in the figure 3 of the manuscript is described in figure S3. The ferromagnetic nanowires are 50nm wide and 20nm thick. The overlap with the non-magnetic spacer is 50nm long. Note that the last 25nm of the wires are of triangular shape as well, in order to avoid domain wall and inhomogeneous magnetization at the edge of the wire. The different distances inter-electrodes (L_1 and L_2) are represented on the figure S3. They are 100nm and 50nm long, respectively. The length of the spacer is 200nm, while its width is 250nm. Its thickness is 60nm.

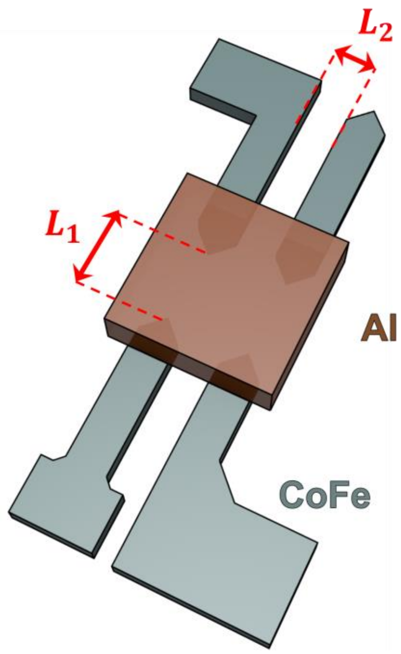

Figure S3: Schematic the CoFe and Al parts of the device. The different distances inter-electrodes are also represented.

MAGNETIZATION DETECTION OF A NANODISK (GEOMETRY DETAILS)

The nanodevice presented in the figure 4 of the manuscript is described in figure S4. The ferromagnetic nanowires are 50nm wide and 15nm thick. The ferromagnetic nanodisk has 160nm wide diameter and is 15nm thick. The non-ferromagnetic nanowires are 50nm wide and 60nm thick. The distance between an external electrode and the nanodisk is 300nm center-to-center (L) and is represented on the figure S4.

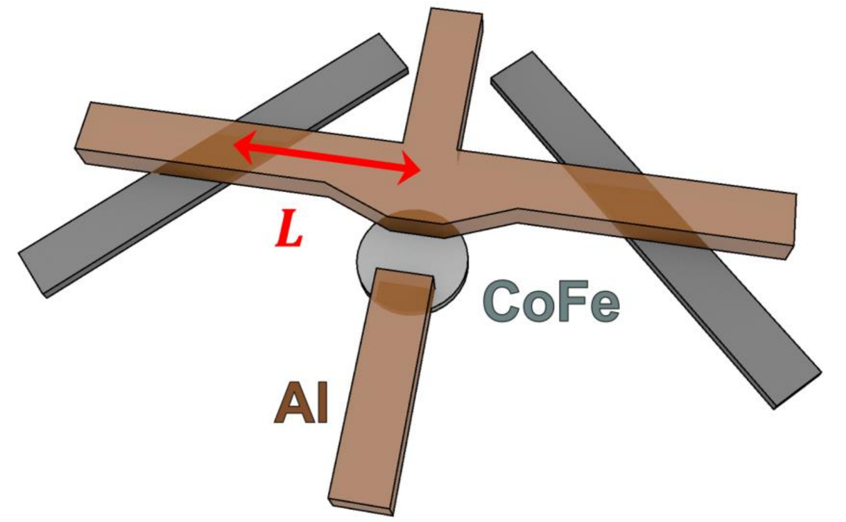

Figure S4: Schematic the CoFe and Al parts of the device. The distance between one external and the central electrodes is also represented.

SIMULATION DETAILS

The simulations have been performed using the geometry shown in figure S2. The meshing has been realized using the GMSH[1] software. The mesh size are lower near the interfaces, where the spin and charge currents are likely to be less homogeneous, as seen in figure S5c below.

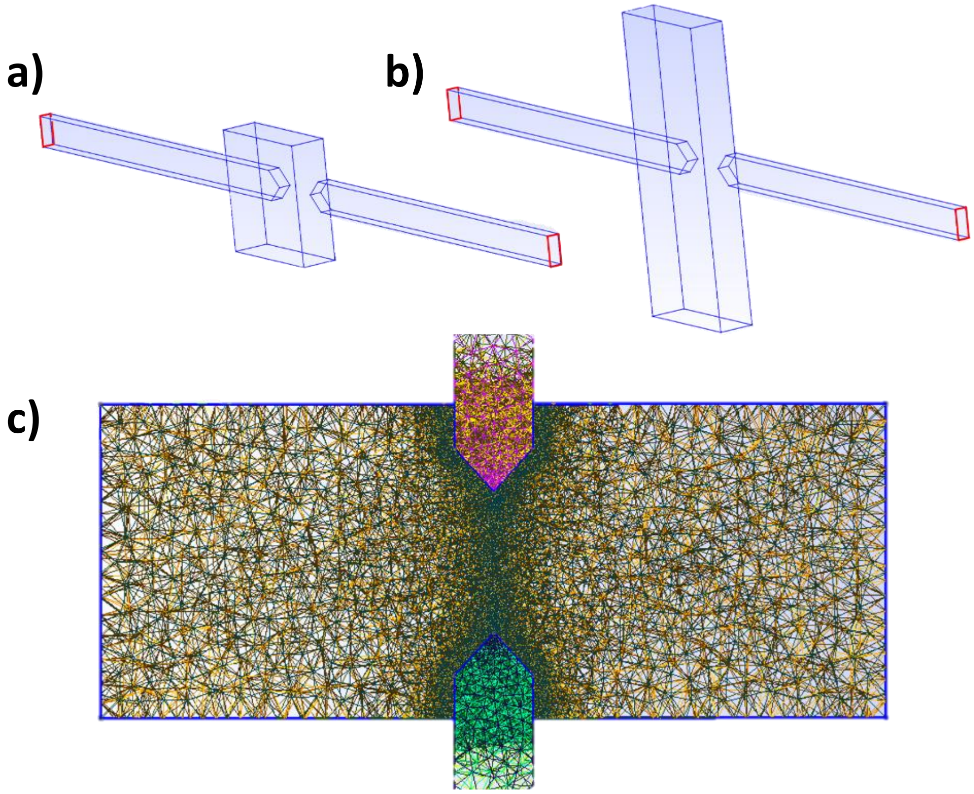

Figure S5: a) and b) Examples of three dimensional geometries. The corresponding devices have 200nm wide and 500nm wide spacers, respectively. The area circled by red edges are the surfaces where the electrical current is injected and where the calculated electrical potential has been averaged to extract the spin signal levels. The ferromagnetic wires have been set to be long, to ensure that the out-of-equilibrium state at the ferromagnetic/non-magnetic interfaces does not influence the potentials in the probing area (i.e., in the red-circled areas $\mu^{\uparrow} \approx \mu^{\downarrow} \approx \mu$*). c) Example of tetraedron-based mesh used for the finite element method simulations. The triangles are randomly oriented, to avoid creating any artifact.*

The finite element method simulations have been performed using GETDP[2] as a solver. The simulations are based on a two spin-current drift-diffusion model resulting from the Valet-Fert model. The electrochemical potentials of each spin population ($\mu^{\uparrow}$ and $\mu^{\downarrow}$) are thus calculated everywhere in each volume. The probed difference of potentials is taken as the difference between the electrical potentials ($\mu = \frac{\mu^{\uparrow}+\mu^{\downarrow}}{2}$) at the ends of the nanowires (areas circled by red edges on the figure S5a and S5b). For each width, two different simulations have been performed, with the relative magnetizations of the electrodes being parallel or antiparallel. Then the GMR spin signal is calculated as the difference between the voltages obtained in the parallel and in the antiparallel configurations. The calculated spin and charge currents field have been examined, to verify that the simulations reproduce the expected physical behaviors and that they are free from singularities. An example is presented in figure S6.

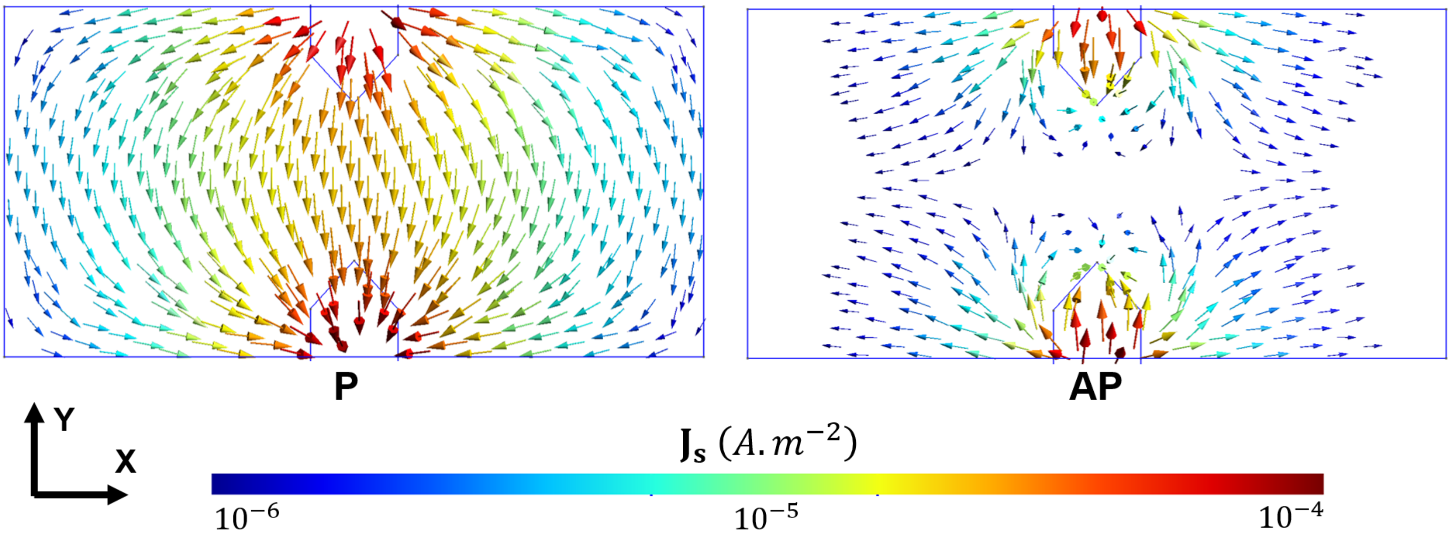

Figure S6: Examples of a spin current field, for a device with a 400nm wide spacer (top view). On the left picture, the magnetic state is parallel. The spin accumulations generated at the interfaces have opposite signs, so that the spin current flows mostly along the Y direction, from one interface to the other. On the right picture, the magnetic state is antiparallel, and the spin accumulations are of same sign. The spin accumulation thus relaxes in every direction. Note that the spin current field in the ferromagnetic element have not been drawn, for the sake of clarity. As all the equations used for the simulations are linear, the current density values correspond to an injected current set at 1 A.

---

[1] Geuzaine, C., & Remacle, J. F. Gmsh: A 3-D finite element mesh generator with built-in pre-and post-processing facilities. *International Journal for Numerical Methods in Engineering*, **79**, 1309-1331 (2009)

[2] Geuzaine, C. GetDP: a general finite-element solver for the de Rham complex. *PAMM*, **7**, 1010603-1010604 (2007)
